# Major Depressive Disorder Alters Perception of Emotional Body Movements

**DOI:** 10.3389/fpsyt.2014.00004

**Published:** 2014-01-20

**Authors:** Morten Kaletsch, Sebastian Pilgramm, Matthias Bischoff, Stefan Kindermann, Isabell Sauerbier, Rudolf Stark, Stefanie Lis, Bernd Gallhofer, Gebhard Sammer, Karen Zentgraf, Jörn Munzert, Britta Lorey

**Affiliations:** ^1^Cognitive Neuroscience Group, Center for Psychiatry and Psychotherapy, Justus Liebig University Giessen, Giessen, Germany; ^2^Institute for Sports Science, Justus Liebig University Giessen, Giessen, Germany; ^3^Bender Institute of Neuroimaging, Justus Liebig University Giessen, Giessen, Germany; ^4^Institute for Sports Science, University of Münster, Münster, Germany; ^5^Central Institute of Mental Health, Medical Faculty Mannheim, Heidelberg University, Mannheim, Germany

**Keywords:** major depressive disorder, emotion perception, point-light displays, social cognition, embodiment, body movements, kinematics

## Abstract

Much recent research has shown an association between mood disorders and an altered emotion perception. However, these studies were conducted mainly with stimuli such as faces. This is the first study to examine possible differences in how people with major depressive disorder (MDD) and healthy controls perceive emotions expressed via body movements. Thirty patients with MDD and thirty healthy controls observed the video scenes of human interactions conveyed by point-light displays (PLDs). They rated the depicted emotions and judged their confidence in their rating. Results showed that patients with MDD rated the depicted interactions more negatively than healthy controls. They also rated interactions with negative emotionality as being more intense and were more confident in their ratings. It is concluded that patients with MDD exhibit an altered emotion perception compared to healthy controls when rating emotions expressed via body movements depicted in PLDs.

## Introduction

As social beings, it is important for us to recognize and properly assess the emotions of our conspecifics, so that we can adapt our own behavior accordingly. This can be advantage to us, because we would approach people who seem to be in a good mood or are sad and needing comfort or help, but avoid contact with those who are angry, threatening, or dangerous in order to protect ourselves. We learn about the meaning of emotions by observing others’ emotions and the behavior that accompanies them. Implicit and explicit processes of mentalization such as imitating and mirroring emotions play an important role in helping us to judge our interaction partners’ emotional state and intentions accurately, so that we can predict their prospective behavior and respond to it appropriately (1–3).

Past research has indicated repeatedly that people with mood disorders such as major depressive disorder (MDD) exhibit alterations and deficits in areas of social cognition, empathy, emotion processing, and emotion perception (4–7) “irrespective of age of onset/duration of illness, task type, diagnosis, sex, and hospitalization status” (8). More precisely, people with MDD show a negative response bias, pay greater attention, attend more selectively, and show stronger emotional reactions when processing emotional and particularly negative stimuli; and, in addition, they remember negative stimuli better than positive stimuli (4, 9–14). Forty years ago, Beck already formulated his cognitive theory of depression postulating that people with depression show a negative biased information processing and an altered negative biased view of the world compared to healthy controls (15–17).

Additionally, on a neural level, altered neural activation patterns have been reported in those areas responsible for processing emotional stimuli (limbic structures, prefrontal regions such as the ventromedial prefrontal cortex) and higher cognitive processes (dorsolateral prefrontal cortex). These have been associated especially with the onset and persistence of mood disorders such as MDD (18, 19).

Most research on this topic has focused on emotions expressed via facial expression and prosody. This neglects an important human emotion expressing system: the human body, that is, body language and body movements. Human body movement can also convey emotions, and observers can infer the emotional state of an individual or interacting partners from movements even when they are at a distance and the faces of the interacting persons are not clearly visible (20–25). Like the face, the body is a source of information on a person’s internal emotional state. When this emotional state leads to a corresponding body gesture, this gesture, in turn, functions as a signal to any observer. The observers’ reactions to this signal will be very fast and may help to protect them, even without seeing the other person’s facial expression. Emotional body movements not only just provide information on the threat, as a facial expression does, but also a direct cue regarding an adequate behavioral response (26). Bearing in mind that people with MDD show altered emotion perceptions of facial expressions, and bearing in mind that not only the face but also the entire body – especially body movements – express emotions, it would seem important to investigate whether and how mood disorders such as MDD influence the perception of emotions in body movements in order to increase our scientific knowledge and better understand the complex process of emotion perception.

To investigate the perception of emotional body movements with a complete exclusion of facial information and other distracting variables, this study exploits the advantages of point-light displays (PLDs). Since the seminal work of Johansson in 1973 (20), it is known that human actions can be perceived intuitively even when the only information available to an observer comes from just a few points representing the joints of the body. Experimentally, such research is implemented with the so-called point-light technique. This method records the kinematics of a few dots placed on a model’s body and uses these to reconstruct PLDs. PLDs have been applied to study not only gait direction or gender recognition (27, 28) but also how human movements represent an individual’s emotional state. The latter research has revealed that emotions can be detected reliably even when no facial expression is seen and emotion perception and recognition can draw only on the biological movement and its kinematics (29). The advantage of using such highly simplified PLDs is that they provide only kinematic movement information. This ensures that the perception process is not influenced by confounding variables in the stimulus material such as attractiveness, sympathy, and the cultural aspects found in the complex and natural stimuli of, for example, faces or whole-body presentations (30).

Against the background of previous research on emotion perception among people with MDD, we studied whether people with MDD would show altered emotion perception of the emotional body movements conveyed by PLDs. We hypothesized that (a) patients with MDD would show a negative bias when rating the emotional valence of the depicted interactions compared to healthy controls; (b) patients with MDD would perceive negative interactions more intensely than positive interactions and healthy controls; and (c) patients with MDD would differ from controls in how confident they were about their ratings of emotional valence.

## Materials and Methods

### Ethical statement

The study was specifically approved by the local ethics committees (local ethics commissions, Department of Psychology and Sports Science, Department of Medicine, Justus Liebig University Giessen), and all participants gave their informed written consent in accordance with the Declaration of Helsinki. All participants gave written consent to participate in the study.

### Participants

The total sample consisted of 60 middle-aged adults: 30 patients receiving treatment at the Centre of Psychiatry and Psychotherapy at the university hospital of Justus Liebig University Giessen and 30 healthy controls.

The 30 patients (16 female, mean age = 50.5 years, SD = 11.25) were diagnosed with MDD according to DSM-IV criteria. At the time of testing, 21 patients were taking antidepressants; 9 patients, a combination of drugs (antidepressant and/or sedative and/or antipsychotic drug and/or mood stabilizer). Seven patients met the criteria for another mental disorder: anxiety disorder (*n* = 2), post-traumatic stress disorder (*n* = 1), eating disorder (*n* = 1), persistent somatoform pain disorder (*n* = 1), anxious avoidant personality disorder (*n* = 1), and dependent personality disorder (*n* = 1).

Diagnoses of MDD were conducted by experienced psychiatrists and psychologists. Patients with present or previous neurological disease or trauma, alcohol or drug dependence, acute and chronic psychotic disorders, bipolar disorders, as well as other medical conditions that could influence cognitive functioning were excluded.

The 30 age-matched healthy adults (14 female, mean age = 49.9 years, SD = 9.1) were recruited as a control group. Their data has also been used for a preceding study (24). The same exclusion criteria were applied as for patients. In addition, healthy controls were excluded if they had any history of psychiatric or neurological disorders, any history or current use of any psychoactive medication, or a score higher than 13 on the Beck Depression Inventory (BDI-II)

### Producing PLDs

The procedure of creating and validating of stimuli is the same as that described by Lorey et al. (24). Seven pairs of two actors provided the movements for PLDs. Each pair was asked to perform an interaction portraying one of the following four emotions: anger, sadness, joy, and love. Interactions with anger and sadness were pooled in the category “negative” and interactions with love and joy were pooled in the category “positive.” Prior to acting, both actors were given a script instructing them to perform the same emotion in order to produce a behavioral pattern that was as symmetrical as possible. Actors were asked to act out the emotion immediately. They were completely free to express their emotions in whatever way they liked – for example, by overt symbolic gestures. At least four clips of each pair and each emotional scene were produced. In addition, for each of the dyadic PLDs (scene with two actors: dyad), a monadic PLD version was created consisting of the dots of one of the two individuals alone (scene with one actor: monad). Apart from this, they still displayed the same emotion with the same movements. This resulted in a corpus of 96 recordings with 8 recordings for each category (monad vs. dyad × positive vs. negative × three difficulty Levels, see section below). The factors difficulty (easy, medium, difficult) and Social context (monad, dyad) were used in another part of this project and are therefore not analyzed and discussed here.

**Figure 1:**
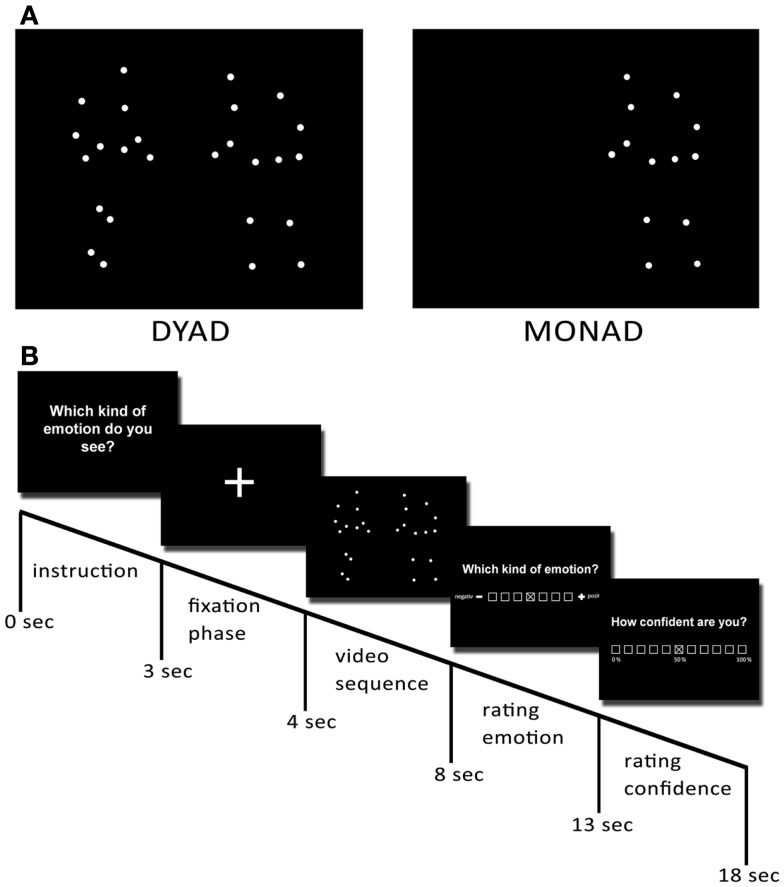
**Preparation of stimuli**. To create the point-light displays, 13 reflective markers were attached to an actor’s head, shoulders, elbows, wrists, hips, knees, and ankles. They were then tracked using a Vicon motion-capture system. **(A)** Examples of dyadic and monadic point-light displays. **(B)** Temporal structure of one trial of the experiment.

All interactions were recorded with a 12-camera VICON MX system (Oxford Metrics, Oxford, England) operating at 100 Hz. Thirteen reflective markers were attached to defined anatomical landmarks on the upper body (including the shoulders, the elbow joints, the wrists, and the forehead) and the lower body (including the hips, the knee joints, and the ankles) of each actor (Figure 1). After capturing, data post-processing was conducted with Nexus 1.5.2 (Vicon Motion Systems, Oxford, England) in order to calculate 3-D coordinates of the markers. The video files were created in a two-step process using Matlab software (MathWorks, Natick, MA, USA). First, for each point in time, the 3-D coordinates of the 13 markers were plotted as white spheres on a black background. Then, the frames of the captured scenes were rendered as audio–video interleaved (avi) movie files at a frame rate of 25 Hz. For each scene, video files with a duration of 4 s were created from a front view. In all presented PLDs, the dots appeared white against a black background at an approximate viewing distance of 50 cm.

### Stimuli: Validation and determination of item difficulty

Prior to the experiment, an index of item difficulty was determined for all recorded PLDs in order to separate the recordings into three classes (easy, medium, and difficult to recognize). We asked 30 participants who did not participate in the present study to evaluate the negativity or the positivity of the emotions displayed in the videos in a forced-choice paradigm. The three categories of item difficulty were created by calculating the percentage of people who agreed on the depicted emotion of the video scene. Thus, easy videos were defined by a consensus of 91–100%; medium videos, by a consensus of 71–90%; and difficult videos, by a consensus of 50–70%.

### Procedure

Prior to or after the actual experiment, participants attended a control session, so that experimenters could assess data ensuring that all participants were able to recognize movements from PLDs. They were given control stimuli depicting sports movements such as juggling and basketball, and asked to give a brief definition of each movement as quickly as possible. One-half of the participants started with the experiment and the other-half with the control session in order to control for sequence effects.

The experiment presented a series of 96 video trials (8 sequences per condition: monads vs. dyads × negative vs. positive emotions × three difficulty levels). Conditions were presented in a pseudorandomized order counterbalanced across participants. Each trial started with a fixation phase (1 s), followed by the instruction (3 s) and the respective video sequence (4 s). After observing this sequence, participants were asked to assess the depicted emotional valence of the videos on a seven-point scale ranging from 1 (negative) to 7 (positive) with 4 marking the neutral center of the scale. The position of the valence label (negative) was altered from the left to the right side for one-half of the participants and from right to left for the other half of the participants. After each valence rating, participants were asked to report how confident they were about their rating on an 11-point scale ranging from 1 (0% confidence) to 11 (100% confidence).

### Data analysis and statistics

To control for sequence effects, prior to testing scale, labels (“negative” and “positive”) were reversed for one-half of the participants. Subsequent, in order to conduct statistical analysis of differences in rated valence, data for half of the participants had to be reversed again, so that scores of 1–3 always reflected a negative rating and scores from 5 to 7 a positive rating. For statistical analysis, we calculated mean scores for each rating and each experimental condition. Mean scores for the perceived valence were calculated by summing up all responses from the 7-point scale (most negative = 1, most positive = 7) and dividing the sum by the number of displayed videos. Intensity of ratings was operationalized as the extent participants used to rate closer to the maximum value of the 7-point scale, to say that intensity was coded higher when the rating was closer to the ends of the scale. To create mean scores of intensity of ratings, all scores on the 7-point scale for ratings of perceived valence of negative videos were reversed (1 into 7, 2 into 6, and so on), to receive scores, which were comparable to scores for positive videos, i.e., for both kinds of videos (negative and positive) higher scores meant higher intensity of ratings. Mean scores for the confidence were calculated by summing up all responses from the 11-point scale (1 = 0% confidence to 11 = 100% confidence) and dividing the sum by the number of displayed videos.

To explore the potential differences between patients with MDD and healthy controls in perceiving emotional valence, perceived intensity of emotions, and the confidence in emotion perception, we computed three repeated-measures ANOVAs for perceived valence, intensity, and confidence to examine the effects of the depicted emotion of interaction (positive vs. negative), the social context (monads vs. dyads), the difficulty of videos (easy, medium, difficult), and group as a categorical between-group factor (Table 1).

**Table 1:** **Statistical data of depicted emotion × social context × difficulty repeated-measures ANOVA for rating of valence, intensity of ratings, and confidence rating in ratings**.

	df	*F*	η^2^	*p*
**RATING OF EMOTIONAL VALENCE**				
Group (between-group factor)	1, 58	8.60	0.13	0.005*
Depicted emotion	1, 58	1190.02	0.95	0.000*
Depicted emotion*group	1, 58	8.50	0.13	0.005*
Social context	1, 58	0.11	0.00	0.74
Social context*group	1, 58	0.32	0.00	0.58
Difficulty	2, 116	13.93	0.19	0.000*
Difficulty*group	2, 116	1.95	0.03	0.15
Depicted emotion*social context	1, 58	420.38	0.88	0.000*
Depicted emotion*social context*group	1, 58	0.11	0.00	0.75
Depicted emotion*difficulty	2, 116	256.23	0.82	0.000*
Depicted emotion*difficulty*group	2, 116	0.16	0.00	0.85
Social context*difficulty	2, 116	23.10	0.29	0.000*
Social context*difficulty*group	2, 116	0.54	0.01	0.58
Depicted emotion*social context*difficulty	2, 116	9.54	0.14	0.000*
Depicted emotion*social context*difficulty*group	2, 116	0.74	0.01	0.48
**INTENSITY OF RATINGS**				
Group (between-group factor)	1, 58	8.50	0.13	0.005*
Depicted emotion	1, 58	0.09	0.00	0.77
Depicted emotion*group	1, 58	8.60	0.13	0.005*
Social context	1, 58	420.37	0.88	0.000*
Social context*group	1, 58	0.11	0.00	0.75
Difficulty	2, 116	256.23	0.82	0.000*
Difficulty*group	2, 116	0.16	0.00	0.85
Depicted emotion*social context	1, 58	0.11	0.00	0.74
Depicted emotion*social context*group	1, 58	0.32	0.00	0.32
Depicted emotion*difficulty	2, 116	13.93	0.19	0.000*
Depicted emotion*difficulty*group	2, 116	1.95	0.03	0.15
Social context*difficulty	2, 116	9.54	0.14	0.000*
Social context*difficulty*group	2, 116	0.75	0.01	0.48
Depicted emotion*social context*difficulty	2, 116	23.10	0.29	0.000*
Depicted emotion*social context*difficulty*group	2, 116	0.54	0.00	0.58
**CONFIDENCE IN RATING**				
Group (between-group factor)	1, 58	0.20	0.00	0.66
Depicted emotion	1, 58	25.51	0.31	0.000*
Depicted emotion*group	1, 58	4.23	0.07	0.04*
Social context	1, 58	175.51	0.75	0.000*
Social context*group	1, 58	0.13	0.00	0.72
Difficulty	2, 116	95.311	0.62	0.000*
Difficulty*group	2, 116	1.53	0.03	0.22
Depicted emotion*social context	1, 58	1.15	0.02	0.29
Depicted emotion*social context*group	1, 58	0.06	0.00	0.81
Depicted emotion*difficulty	2, 116	2.58	0.04	0.08
Depicted emotion*difficulty*group	2, 116	0.37	0.00	0.69
Social context*difficulty	2, 116	14.30	0.20	0.000*
Social context*difficulty*group	2, 116	0.80	0.01	0.45
Depicted emotion*social context*difficulty	2, 116	21.43	0.27	0.000*
Depicted emotion*social context*difficulty*group	2, 116	0.39	0.00	0.68

ANOVA, *p < 0.05.

All statistics were calculated using SPSS software (Versions 19 and 20), and an alpha level of 0.05 was used for all statistical tests.

## Results

### Control data

#### Control session: biological motion recognition test

Participants were able to identify each of the actions reliably and far above chance level. On average, 93.24% (range: 67–100%) of classifications was correct. Implementing the control session either before or after the main experimental session did not result in any significant difference in the ratings of either emotional valence, *t*(58) = 0.62, *p* > 0.05 or confidence, *t*(58) = 0.94, *p* > 0.05.

#### Position of valence label during valence rating

Position of the valence labels (negative and positive left or right) did not produce systematically different valence ratings, *t*(58) = 0.80, *p* > 0.05, or confidence ratings, *t*(58) = 0.15, *p* > 0.05, in the main trail.

### Influence of group on rated valence (negative or positive)

**Figure 2:**
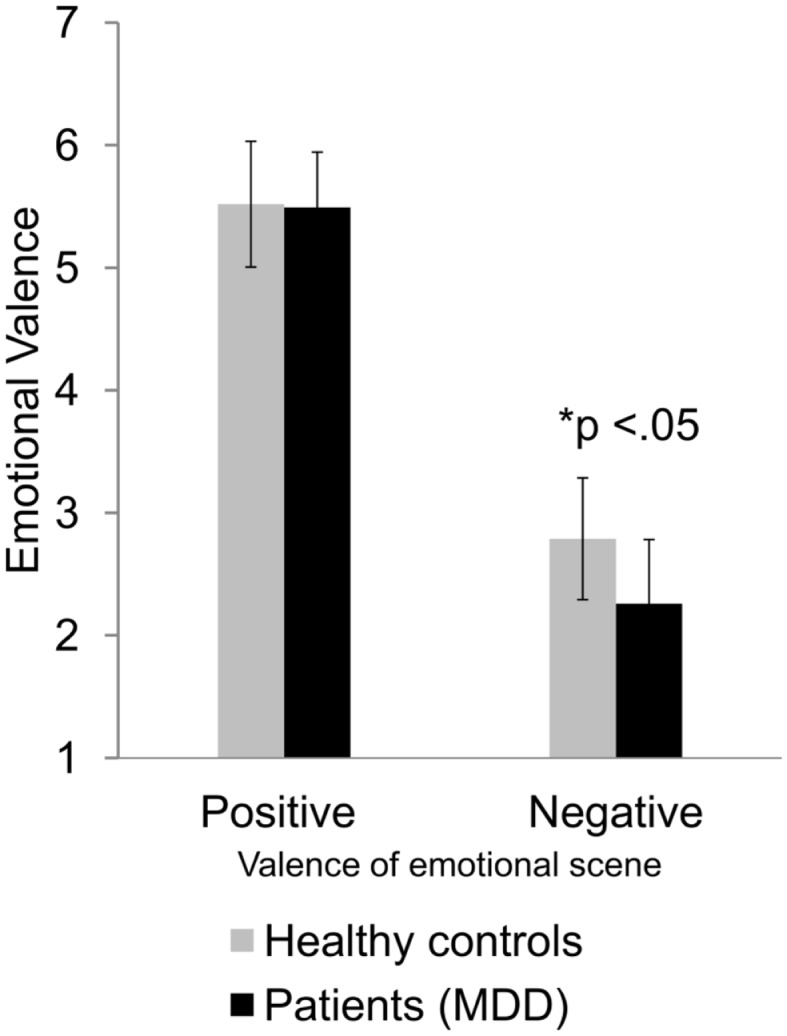
**Differences in rating of valence of patients with MDD and healthy controls for positive and negative emotional interactions**. Average valence ratings and their standard deviations are displayed as a function of participant group (healthy controls vs. patients with MDD) and valence of depicted emotional scene (positive vs. negative). The difference is significant at the 0.05 level.

There was a significant effect of group membership on rating of perceived emotional valence, *F*(1, 58) = 8.60, *p* < 0.05, η^2^ = 0.13. Patients with MDD rated depicted scenes more negatively than healthy controls. Interestingly, the significant two-way interaction between depicted emotion and group revealed that patients with MDD rated only negative emotional interactions more negatively than healthy controls but not positive emotional interactions, *F*(1, 58) = 8.50, *p* < 0.05, η^2^ = 0.13 (Figure 2). See Table 1 for all results of the repeated-measures ANOVAs. None of the two-way, three-way, or four-way interactions (depicted emotion, social context, difficulty) with group turned significant.

### Influence of group on intensity of ratings

**Figure 3:**
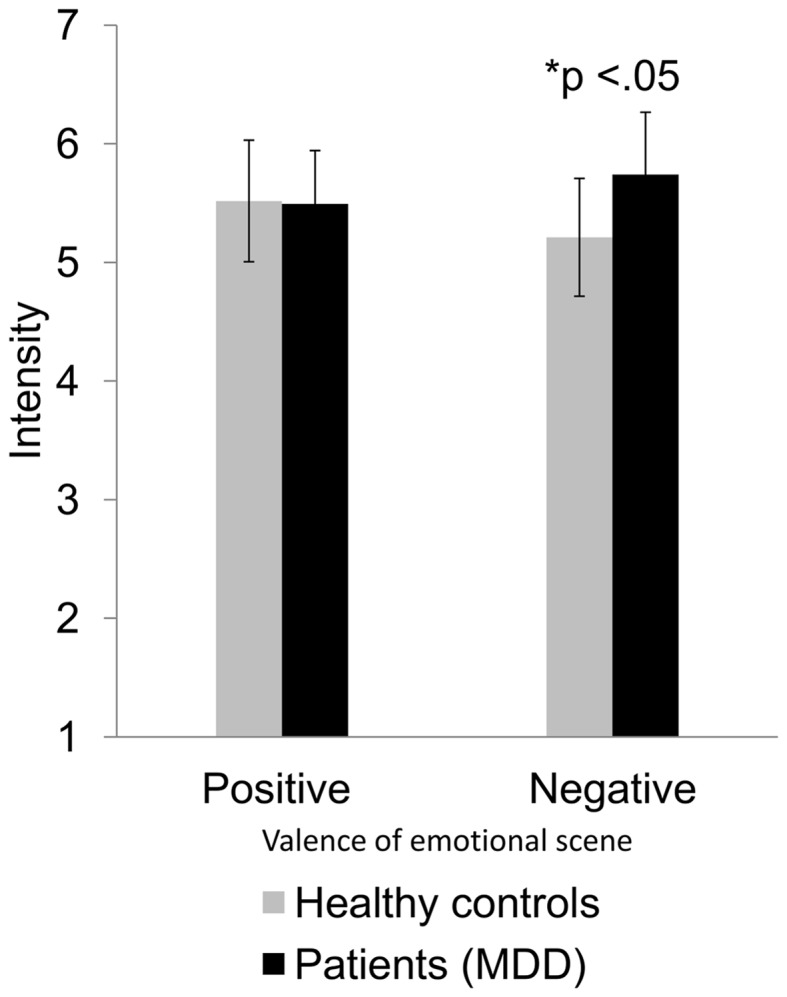
**Differences in rating intensity of patients with MDD and healthy controls for positive and negative emotional interactions**. Average intensity ratings and their standard deviations are displayed as a function of participant group (healthy controls vs. patients with MDD) and valence of depicted emotional scene (positive vs. negative). The difference is significant at the 0.05 level.

Regarding the intensity of participants’ ratings on depicted valence, ANOVAs again revealed a significant main effect of group, *F*(1, 58) = 8.50, *p* < 0.05, η^2^ = 0.13. Similar to the prior analysis of rated valence, the two-way interaction between group and depicted emotion attained significance, *F*(1, 58) = 8.60, *p* < 0.05, η^2^ = 0.13 (Figure 3), showing that the difference occurred only when the depicted emotion of the emotional interactions was negative, but not when it was positive. Patients with MDD rated emotional negative interactions more intensely (*M* = 5.74, SD = 0.52) than healthy controls (*M* = 5.21, SD = 0.49). None of the two-way, three-way, or four-way interactions (depicted emotion, social context, difficulty) with group turned significant.

### Influence of group on confidence in ratings

**Figure 4:**
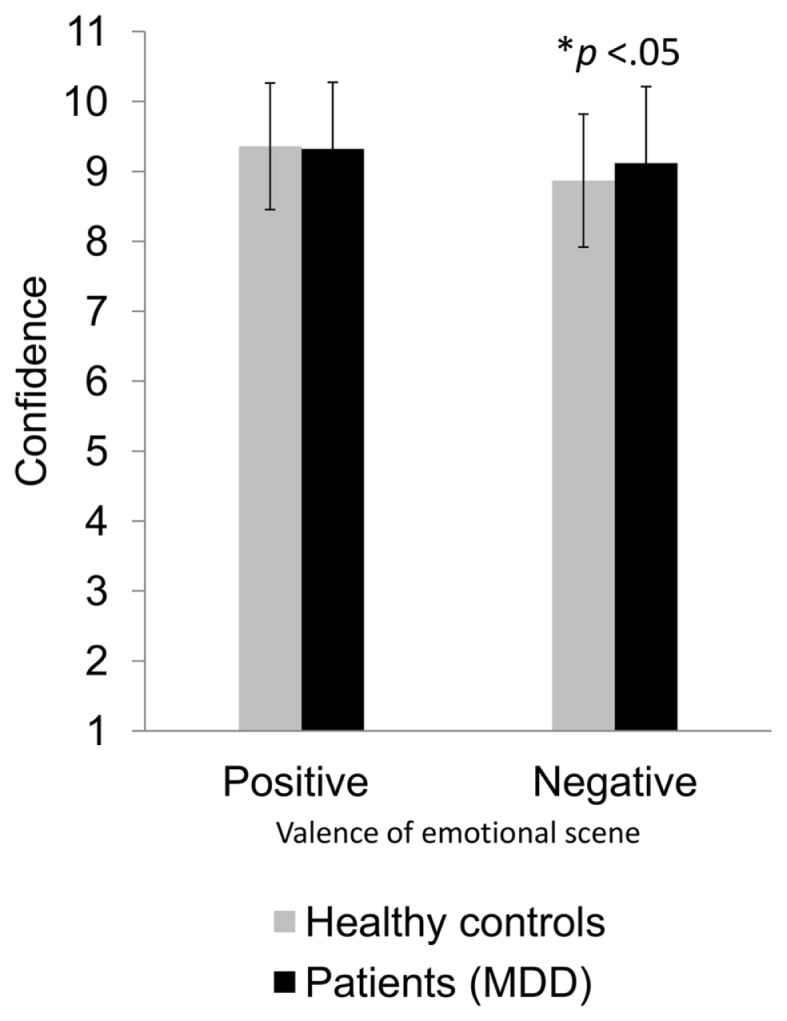
**Differences in rating confidence of patients with MDD and healthy controls for positive and negative emotional interactions**. Average confidence ratings and their standard deviations are displayed as a function of participant group (healthy controls vs. patients with MDD) and valence of depicted emotional scene (positive vs. negative). The difference is significant at the 0.05 level.

There was no significant main effect of group membership on confidence in the rating of emotional valence *F*(1, 58) < 1, ns, but a significant two-way interaction between group and valence, *F*(1, 58) = 4.22, *p* < 0.05, η^2^ = 0.07 (Figure 4). When the emotional scene was positive, patients with MDD rated valence just as confidently as healthy controls. In contrast, when the valence was negative, patients with MDD were more confident about their perceptions and ratings than healthy controls. None of the two-way, three-way, or four-way interactions (depicted emotion, social context, difficulty) with group turned significant.

Interestingly, even though they were not the main subject of this study, depicted emotion was the only one of the three design factors to produce a group difference. Neither difficulty of stimuli nor the number of interaction partners resulted in group differences in rating either valence, intensity of ratings, or confidence in ratings. Furthermore, the statistical analysis revealed neither a confounding effect of the variable gender nor an effect of comorbidity on all outcome variables.

## Discussion

The present study investigated differences in the perception of emotional body movements in patients with MDD compared to healthy controls. Point-light video scenes of human interactions including only emotional body movements with no information on facial expression were used to determine possible differences. Despite the importance of body language in social functioning, this is the first study to examine the relationship between affective disorders and the perception of emotions expressed through body movements. It is also the first study to use PLDs to investigate the differential effects of MDD on emotion perception, intensity of emotion, and confidence in rating the emotions present in observed interactions. These displays of either one or two persons interacting emotionally provided exclusively kinematic movement information and no facial expression. As predicted, the results show altered emotion perception in patients with MDD compared to healthy controls. More precisely, a negative bias when rating negative emotional body movements emerged. People with MDD reported a higher intensity when perceiving negative emotional movements and were more confident about their ratings of negative emotional interactions than healthy controls.

Just like research on emotion perception of facial expression in persons with MDD (4, 9–14), our findings show the same negative response bias toward interactions containing negative emotional body movements in patients with MDD. Within this patient group, interactions with aggressive, angry, or sad content led to more negative evaluations than in a group of healthy controls. As in previous research, this negative response bias occurred only when the emotional content of the interactions was negative but not when it was positive. The results can be interpreted in line with Beck’s cognitive theory of depression, in which people with depression are considered to hold dysfunctional attitudes that are activated by stressful events and result in a negative cognitive bias (17). This then leads to the following interesting questions: how does this negative bias influence social behavior? Which direction does this bias take? Is it advantageous or disadvantageous to show this bias? These questions are especially relevant when – as in the present study – the bias occurs only when perceiving negative emotional interactions. Further research could examine whether this bias influences social approach and avoidance behavior, and how this more negative perception influences state and mood and, therefore, the symptoms and maintaining of MDD.

Regarding the intensity of responses, we found more intense responses to negative emotional interactions from people with MDD compared to healthy controls. This is similar to the stronger emotional reaction to negative stimuli among depressed people reported by Persad and Polivy (31). It could be discussed within the context of the negative potentiation hypothesis proposing that potentiated emotional reactivity to negative emotional stimuli is elicited by negative mood or emotional states (32, 33). However, it has to be pointed out here that most studies on the negative potentiation hypothesis did not investigate the perceived intensity of observed interpersonal interactions. The subjective emotional reactivity to these interactions might well differ from the judgment of their intensity. In contrast, no difference was found for positive emotional interactions. This might be interpreted and discussed in light of previous research indicating that the higher salience of negative emotions combined with negative bias leads to higher perceived intensity of such interactions, whereas positive interactions are less salient. As a result, their emotional intensity may well tend to be missed.

The effect that people with MDD are more confident about rating the valence of negative emotional interactions could be interpreted in line with previous findings reporting an increased vigilance and selective attention toward negative emotions in this patient group (4, 12, 34, 35). People with depression may be more vigilant and pay more attention when perceiving negative emotional interactions, and therefore more confident when rating their valence. They may be more familiar with experiencing negative stimuli and therefore more confident about such perceptions.

The present data demonstrate a differentiated emotion perception in people with MDD compared to healthy controls. It is important to mention that emotions were perceived on the basis of emotional body movements. Of course, facial expressions have the function of imparting information about a person’s emotional state. However, body gestures do more than just that; they can also deliver cues on how best to behave in a certain situation. The negatively biased misinterpretation of these “negative” behavioral cues can lead an observer to withdraw, because the expected consequences of approaching or staying are interpreted more negatively than they really are (26).

Especially, a negative bias could discourage patients with MDD from establishing social contacts, particularly when they observe another person’s emotional body movements from a distance and perceive and misinterpret those as negative or more negative than they actually are. This could lead them to avoid approaching this person. Without active attempts to get in touch with other people, it is difficult to establish or broaden a social network that could, in turn, lead to greater social support, a sense of belonging, and well-being. Hence, becoming aware that one’s judgments are based on a false interpretation of the emotion perception of body movements could be another step toward reconsidering and maybe altering these judgments in order to avoid their negative consequences (36, 37).

This is where a training could help to improve emotion perception and reduce negative misinterpretation and thereby eventually help to establish more social contacts (38). In particular, a meta-cognitive training could help to make people with MDD aware of these cognitive processes and enable them to gradually make perception more realistic and accurate (38, 39).

## Conclusion

This is the first study to investigate differences between patients with MDD and healthy controls when perceiving emotions expressed via body movements and body movements conveyed by PLDs. First, when perceiving emotions from body movements, patients with MDD show a similar negative bias to that shown when judging facial emotional expressions. Second, they perceive and judge negative emotional interactions more intensely and more confidently than positive emotional interactions. Hence, our data support other similar findings on the altered emotion perception of patients with MDD compared to healthy controls, thereby extending research on emotion perception to the domain of emotional body movements and kinematics. These first results expand our knowledge about the differentiated emotion perception to be found in depressive patients. Further studies could contribute to our understanding of approach and especially avoidance behavior in social interactions. Such insights might well find their way into a meta-cognitive training that could help to reduce the maintaining factors of MDD such as the avoidance of social contacts due to negative perception bias.

## Conflict of Interest Statement

The authors declare that the research was conducted in the absence of any commercial or financial relationships that could be construed as a potential conflict of interest.

## References

[1] Banziger T Grandjean D Scherer KR Emotion recognition from expressions in face, voice, and body: the Multimodal Emotion Recognition Test (MERT). Emotion (2009) 9:691–704 10.1037/a0017088 19803591

[2] Derntl B Habel U Deficits in social cognition: a marker for psychiatric disorders? Eur Arch Psychiatry Clin Neurosci (2011) 261(Suppl 2):S145–9 10.1007/s00406-011-0244-0 21863344

[3] Frith CD Frith U Mechanisms of social cognition. Annu Rev Psychol (2012) 63:287–313 10.1146/annurev-psych-120710-100449 21838544

[4] Leppänen JM Emotional information processing in mood disorders: a review of behavioral and neuroimaging findings. Curr Opin Psychiatry (2006) 19:34–9 10.1097/01.yco.0000191500.46411.00 16612176

[5] Wang YG Wang YQ Chen SL Zhu CY Wang K Theory of mind disability in major depression with or without psychotic symptoms: a componential view. Psychiatry Res (2008) 161:153–61 10.1016/j.psychres.2007.07.018 18926572

[6] Wright SL Langenecker SA Deldin PJ Rapport LJ Nielson KA Kade AM Gender-specific disruptions in emotion processing in younger adults with depression. Depress Anxiety (2009) 26:182–9 10.1002/da.20502 18800371 PMC3013355

[7] Romera I Perez V Menchón J Delgado-Cohen H Polavieja P Gilaberte I Social and occupational functioning impairment in patients in partial versus complete remission of a major depressive disorder episode: a six-month prospective epidemiological study. Eur Psychiatry (2010) 25:58–65 10.1016/j.eurpsy.2009.02.007 19553092

[8] Kohler CG Hoffman LJ Eastman LB Healey K Moberg PJ Facial emotion perception in depression and bipolar disorder: a quantitative review. Psychiatry Res (2011) 188:303–9 10.1016/j.psychres.2011.04.019 21601927

[9] Hale WW III Jansen JHC Bouhuys AL Van Den Hoofdakker RH The judgment of facial expressions by depressed patients, their partners and controls. J Affect Disord (1998) 47:63–70 10.1016/S0165-0327(97)00112-2 9476745

[10] Bouhuys AL Geerts E Gordijn MCM Depressed patients’ perceptions of facial emotions in depressed and remitted states are associated with relapse – a longitudinal study. J Nerv Ment Dis (1999) 187:595–602 10.1097/00005053-199910000-00002 10535652

[11] Surguladze SA Young AW Senior C Brebion G Travis MJ Phillips ML Recognition accuracy and response bias to happy and sad facial expressions in patients with major depression. Neuropsychology (2004) 18:212–8 10.1037/0894-4105.18.2.212 15099143

[12] Bourke C Douglas K Porter R Processing of facial emotion expression in major depression: a review. Aust N Z J Psychiatry (2010) 44:681–96 10.3109/00048674.2010.496359 20636189

[13] Naranjo C Kornreich C Campanella S Noël X Vandriette Y Gillain B Major depression is associated with impaired processing of emotion in music as well as in facial and vocal stimuli. J Affect Disord (2011) 128:243–51 10.1016/j.jad.2010.06.039 20663569

[14] Liu W Huang J Wang L Gong Q Chan RCK Facial perception bias in patients with major depression. Psychiatry Res (2012) 197:217–20 10.1016/j.psychres.2011.09.021 22357354

[15] Beck A Thinking and depression: I. Idiosyncratic content and cognitive distortions. Arch Gen Psychiatry (1963) 9:324 10.1001/archpsyc.1963.01720160014002 14045261

[16] Beck A Thinking and depression: II. Theory and therapy. Arch Gen Psychiatry (1964) 10:561 10.1001/archpsyc.1964.01720240015003 14159256

[17] Beck A The evolution of the cognitive model of depression and its neurobiological correlates. Am J Psychiatry (2008) 165:969–77 10.1176/appi.ajp.2008.08050721 18628348

[18] Phillips ML Drevets WC Rauch SL Lane R Neurobiology of emotion perception II: implications for major psychiatric disorders. Biol Psychiatry (2003) 54:515–28 10.1016/S0006-3223(03)00171-9 12946880

[19] Cusi AM Nazarov A Holshausen K Macqueen GM Mckinnon MC Systematic review of the neural basis of social cognition in patients with mood disorders. J Psychiatry Neurosci (2012) 37:100179 10.1503/jpn.100179 22297065 PMC3341408

[20] Johansson G Visual perception of biological motion and a model for its analysis. Percept Psychophys (1973) 14:201–1115820512

[21] Sevdalis V Keller PE Perceiving performer identity and intended expression intensity in point-light displays of dance. Psychol Res (2011) 75:423–34 10.1007/s00426-010-0312-5 20981438

[22] Atkinson AP Vuong QC Smithson HE Modulation of the face-and body-selective visual regions by the motion and emotion of point-light face and body stimuli. Neuroimage (2012) 59:1700–12 10.1016/j.neuroimage.2011.08.073 21924368

[23] Ennis C Egges A Perception of complex emotional body language of a virtual character. Mot Games (2012) 7660:112–21 10.1007/978-3-642-34710-8_11

[24] Lorey B Kaletsch M Pilgramm S Bischoff M Kindermann S Sauerbier I Confidence in emotion perception in point-light displays varies with the ability to perceive own emotions. PLoS One (2012) 7:e42169 10.1371/journal.pone.0042169 22927921 PMC3425494

[25] Barliya A Omlor L Giese MA Berthoz A Flash T Expression of emotion in the kinematics of locomotion. Exp. Brain Res (2013) 225:159–76 10.1007/s00221-012-3357-4 23250443

[26] de Gelder B Towards the neurobiology of emotional body language. Nat Rev Neurosci (2006) 7:242–9 10.1038/nrn1872 16495945

[27] Cutting JE Kozlowski LT Recognizing friends by their walk: gait perception without familiarity cues. Bull Psychon Soc (1977) 9:353–6 10.3758/BF03337021

[28] Brooks A Schouten B Troje NF Verfaillie K Blanke O Van Der Zwan R Correlated changes in perceptions of the gender and orientation of ambiguous biological motion figures. Curr Biol (2008) 18:R728–9 10.1016/j.cub.2008.06.054 18786367

[29] Atkinson AP Dittrich WH Gemmell AJ Young AW Emotion perception from dynamic and static body expressions in point-light and full-light displays. Perception (2004) 33:717–46 10.1068/p5096 15330366

[30] Hoffmann H Kessler H Eppel T Rukavina S Traue HC Expression intensity, gender and facial emotion recognition: women recognize only subtle facial emotions better than men. Acta Psychol (Amst) (2010) 135:278–83 10.1016/j.actpsy.2010.07.012 20728864

[31] Persad SM Polivy J Differences between depressed and nondepressed individuals in the recognition of and response to facial emotional cues. J Abnorm Psychol (1993) 102:358–68 10.1037/0021-843X.102.3.358 8408947

[32] Rottenberg J Gross JJ Gotlib IH Emotion context insensitivity in major depressive disorder. J Abnorm Psychol (2005) 114:627–39 10.1037/0021-843X.114.4.627 16351385

[33] Bylsma LM Morris BH Rottenberg J A meta-analysis of emotional reactivity in major depressive disorder. Clin Psychol Rev (2008) 28:676–91 10.1016/j.cpr.2007.10.001 18006196

[34] Mogg K Bradley BP Williams R Attentional bias in anxiety and depression – the role of awareness. Br J Clin Psychol (1995) 34:17–36 10.1111/j.2044-8260.1995.tb01434.x 7757037

[35] Leyman L De Raedt R Schacht R Koster EHW Attentional biases for angry faces in unipolar depression. Psychol Med (2007) 37:393–402 10.1017/S003329170600910X 17076914

[36] Lara ME Leader J Klein DN The association between social support and course of depression: is it confounded with personality? J Abnorm Psychol (1997) 106:478–82 10.1037/0021-843X.106.3.478 9241950

[37] Hagerty BM Williams RA The effects of sense of belonging, social support, conflict, and loneliness on depression. Nurs Res (1999) 48:215–9 10.1097/00006199-199907000-00004 10414684

[38] Blanch-Hartigan D Andrzejewski SA Hill KM The effectiveness of training to improve person perception accuracy: a meta-analysis. Basic Appl Soc Psych (2012) 34:483–98 10.1080/01973533.2012.728122

[39] Koster EHW Fox E Macleod C Introduction to the special section on cognitive bias modification in emotional disorders. J Abnorm Psychol (2009) 118:1–4 10.1037/a0014379 19222308

